# Nasal Nitric Oxide Levels: Improving the Diagnosis of Primary Ciliary Dyskinesia in Puerto Rico

**DOI:** 10.3390/arm90050050

**Published:** 2022-09-26

**Authors:** Wilfredo De Jesús-Rojas, Francisco Alvarado-Huerta, Jesús M. Meléndez-Montañez, José Muñiz-Hernández, Arnaldo Santos-López, Ricardo A. Mosquera

**Affiliations:** 1 Department of Pediatrics–Anatomy and Neuroanatomy, University of Puerto Rico, Medical Sciences Campus, San Juan, PR 00921, USA; 2 Department of Pediatrics and Basic Science, Ponce Health Science University, Ponce, PR 00716, USA; 3 Department of Natural Science, University of Puerto Rico, Cayey Campus, Cayey, PR 00736, USA; 4 Department of Pediatrics, McGovern Medical School, University of Texas Health Science Center at Houston, Houston, TX 77030, USA

**Keywords:** nasal Nitric Oxide, *RSPH4A*, Primary Ciliary Dyskinesia, founder mutation, cilia, Puerto Rico, genotype, phenotype

## Abstract

**Highlights:**

**What are the main findings?**

- Patients with the *RSPH4A* (c.921+3_921+6del (intronic)) founder mutation have low nNO levels.
- Adding new diagnostic tools such as nNO levels improved the diagnosis of PCD in Puerto Rico.

**What is the implication of the main finding?**

- Levels of nNO should be measured in patients with the *RSPH4A* (c.921+3_921+6del (intronic)) founder mutation as part of the PCD diagnostic algorithm.
- In Puerto Rico, suspected PCD patients should be referred to an accredited PCD center with access to nNO level measurements.

**Abstract:**

Primary Ciliary Dyskinesia (PCD) is a rare genetic disease characterized by motile cilia dysfunction with a prevalence of 1 in 16,309 individuals in Hispanic populations. In Puerto Rico, the prevalence of PCD is unknown. Diagnosis of PCD in Puerto Rico is challenging due to the lack of diagnostic technology. Algorithms for PCD diagnosis include clinical history, genetic testing, ciliary biopsy, and nasal Nitric Oxide (nNO) levels. For the first time, this study successfully implemented and measured the nNO levels in subjects with the *RSPH4A* (c.921+3_921+6del (intronic)) as a diagnostic tool to complement the current algorithm for PCD diagnosis on the island. The nNO level differentiated homozygous subjects with PCD due to the *RSPH4A* (c.921+3_921+6del (intronic)) founder mutation compared to healthy gender-age matched controls and subjects with VUS or negative genetic testing for PCD. The acquisition of state-of-the-art diagnostic tools such as nNO positively impacted and expanded our current PCD diagnostic capabilities in Puerto Rico for our founder genetic mutation. The addition of nNO technology promotes earlier disease screening and recognition for patients with PCD on the island. The access to nNO helped us to properly characterize the PCD diagnosis for patients with the *RSPH4A* (c.921+3_921+6del (intronic)). As a result, our findings will allow us to be part of the national PCD foundation registry and represent Puerto Rican Hispanics in future PCD multicentric clinical trials.

## 1. Introduction

In Puerto Rico, as in many other Latin American countries, Primary Ciliary Dyskinesia (PCD) is challenging to diagnose [1]. Mutations in more than 50 genes have been implicated with PCD, a rare recessive disorder that affects the motile cilia [2]. Around 1 in 16,309 Hispanics are affected by PCD [3,4]. In Puerto Rico, the prevalence of PCD is still unknown, but the presence of a founder genetic mutation (c.921+3_921+6del (intronic)) on the *RSPH4A* gene has previously been described and might influence its prevalence [5]. The *RSPH4A* (c.921+3_921+6del (intronic)) impacts the total number of patients identified as living with PCD on the island. Each associated gene may alter the function of around 200 ciliary structural proteins [6]. As a result, a spectrum of challenging clinical manifestations may result, including neonatal respiratory distress in approximately 80% of the cases despite a full-term gestation, sino-oto-pulmonary disease since birth, infertility, and organ laterality defects (mirror-image) in about 50% of cases [7]. Patients with PCD suffer from a chronic wet cough, recurrent bronchitis exacerbations, development of pulmonary scar tissue, and bacterial pulmonary infections. Without a definitive diagnosis due to the lack and accessibility to PCD diagnostic tools, most patients with suspected PCD will develop pulmonary-related complications, may require supplemental oxygen during adulthood, and eventually meet the criteria for a lung transplant. Due to a lack of resources for lung transplantation in Puerto Rico and the limited access to lung transplantation centers in the United States, having access to new tools for early diagnosis of PCD in Puerto Rico is vital [8]. Lack of the necessary technology and diagnostic tools leads to the misdiagnosis of PCD and, therefore, incorrect management of the disease. As a result, PCD in Puerto Rico is frequently misdiagnosed with other syndromes such as cystic fibrosis, chronic suppurative lung diseases, or bronchial asthma [9].

Diagnostic algorithms for suspected PCD patients recommend considering the clinical history, physical examination, and ancillary testing, including the measurement of nasal Nitric Oxide (nNO) levels [2]. Nitric Oxide (NO) is a signaling molecule produced throughout the human body with metabolic function in the respiratory system [10]. Previous studies demonstrate that nNO levels are significantly lower in subjects with PCD compared to healthy control subjects [11]. For instance, nNO levels serve as an ancillary test for PCD as published in the American Thoracic Society (ATS) and PCD Foundation [12]. No studies have documented the nNO levels in native Puerto Ricans with the *RSPH4A* (c.921+3_921+6del (intronic)) founder mutation. Meta-analyses and prospective cohort studies support nNO testing as a safe, noninvasive, feasible, and accurate PCD diagnostic test [13]. However, nNO testing for PCD diagnosis requires expensive specialized equipment, trained personnel, and standardized operating protocols to be successfully implemented. Due to these requirements, access to accurate nNO testing can be challenging for potential PCD patients, especially in Puerto Rico, where this technology was previously unavailable.

For decades, due to the lack of diagnostic technology for rare genetic pulmonary diseases, PCD diagnosis has been a challenge for physicians in Puerto Rico. Furthermore, most PCD genetic mutations in Puerto Rico are inconclusive for diagnosis due to the high frequency of variants of unknown significance (VUS) in our heterogeneous genetic pool [8,14]. For the first time on the island, this study aims to improve PCD diagnosis by implementing the nNO levels as ancillary PCD testing in Puerto Rico. A cross-sectional study was conducted to measure the nNO levels in Puerto Rican subjects with the *RSPH4A* (c.921+3_921+6del (intronic)) founder mutation and subsequently compared with a gender-age matched control cohort.

## 2. Materials and Methods

### 2.1. Study Design and Recruitment

We conducted a cross-sectional study including patients with confirmed PCD diagnosis, suspected PCD with VUS, suspected PCD with negative genetic testing, and age/sex-matched healthy volunteers. Participants (*n* = 40) included children above five years old and adults recruited from the Pediatric Rare Lung and Asthma Institute and PCD Center in Puerto Rico. Retrospective data from the electronic medical records (EMR) were reviewed to ensure subjects met inclusion criteria. Subject participation included two site visits for nNO measurements, each being at least two weeks apart. Average nNO levels between the two visits were calculated. All participants and/or caretakers provided consent to participate in this study, approved by the Institutional Review Board (IRB) of the University of Puerto Rico, Medical Sciences Campus in San Juan, Puerto Rico (Protocol number: B1730220).

### 2.2. Subjects and Cohorts

Inclusion criteria included subjects of both sexes who are greater than five years of age and have PCD phenotype with confirmatory genetic testing. A PCD phenotype was defined by the United States PCD clinical diagnostic criteria, which include at least two of the following four clinical features: [2]

- Persistent, year-round wet cough that starts before 6 months of age.
- Persistent, year-round nasal congestion that starts before 6 months of age.
- Presence of organ laterality abnormalities.
- Unexplained neonatal respiratory distress in infants born at term.

**Figure 1:**
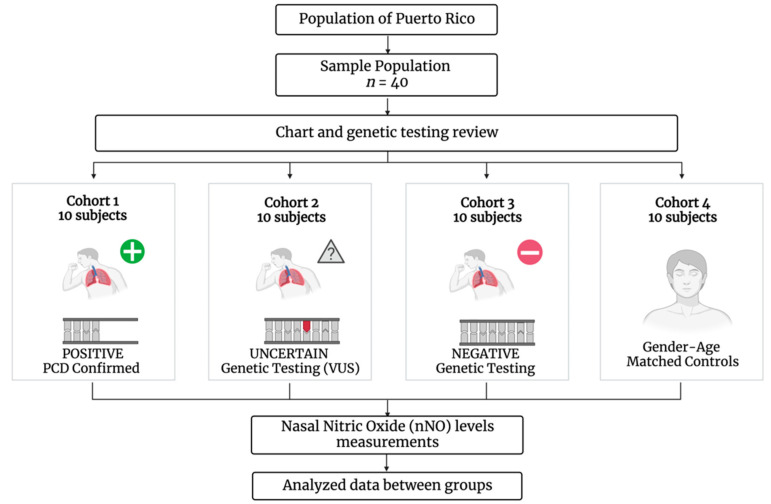
Methodology approach: Stratification of subjects by cohorts for nNO levels measurement analysis.

Patients with PCD phenotype were grouped into three different cohorts for analysis (cohorts 1–3) (Figure 1).

Stratification of all cohorts was completed by considering the individual patient genotype as follows:

- Cohort 1: PCD confirmed subjects: Patients with a confirmed diagnosis of PCD based on clinical phenotype plus diagnosis confirmation by both a nasal biopsy showing microtubular ultrastructure abnormalities by transmission electron microscopy (TEM) and a genetic test positive for bi-allelic pathogenic mutations in *RSPH4A* (c.921+3_921+6del (intronic)) founder mutation.
- Cohort 2: Suspected PCD Subjects with the presence of genetic variants of unknown significance (VUS). Patients with a suspected diagnosis of PCD based on clinical phenotype and the presence of VUS mutations on PCD-related genes.
- Cohort 3: Suspected PCD subjects with negative genetic testing. Patients with a suspected diagnosis of PCD based on clinical phenotype but with negative genetic testing for mutations in the 42 PCD-related genes tested by the Invitae genetic panel.
- Cohort 4: Gender–age-matched healthy controls. Non-atopic, non-smoking patients, defined as patients with no airway or immune problems, no recent significant nasal trauma, no history of systemic infection or inflammation, and no allergies or asthma, were voluntarily invited to participate.

Exclusion criteria for the study included: smokers, history of epistaxis in the past two weeks, acute upper and/or lower infection, presence of an anatomical nasal obstruction, and medical history of cystic fibrosis or immunodeficiencies. PCD patients with *RSPH1* mutations were excluded given reported normal nNO levels in previous studies [15].

### 2.3. Data Collection and Nasal Nitric Oxide (nNO) Levels Measurements

Data collection from EMR included baseline characteristics including age, gender, race, and ethnicity. PCD clinical characteristics, including the history of neonatal respiratory distress, chronic sinusitis, chronic otitis media, laterality defects, hearing loss, and presence of bronchiectasis, were collected and presented. Analysis of the previously ordered genetic test, including analysis of deletions and duplications on 42 PCD-related genes (Genes analyzed: *AK7, ARMC4, C11orf70, CCDC103, CCDC114, CCDC151, CCDC39, CCDC40, CCDC65, CCNO, CEP164, CFAP298, DNAAF1, DNAAF2, DNAAF3, DNAAF4, DNAAF5, DNAH1, DNAH11, DNAH5, DNAH8, DNAH9, DNAI1, DNAI2, DNAJB13, DNAL1, DRC1, GAS8, LRRC56, LRRC6, MCIDAS, NOTCH2, OFD1, PIH1D3, RPGR, RSPH1, RSPH3, RSPH4A, RSPH9, SPAG1, ZMYND10, INVS*) and *CFTR* gene were part of the standard of care for PCD evaluation.

The nNO measurements were performed using a validated chemiluminescence technique following prior published guidelines [13] and EcoPhysics (CLD 88sp Chemiluminescence Nitric Oxide Analyzer, Dürnten, Switzerland) manufacturer recommendations. To complete the nNO level measurement, each patient exhaled through their mouth into a resistor to ensure closure of the soft palate. Criteria for acceptable technique include an exhalation of 20 s or greater with a 3–10 s NO plateau. The nNO measurement was completed on each nostril, and the mean of the findings for each nostril was converted from concentration in part per billion (ppb) to nNO production (nL/min). The cut-off values used were: <77 nL/min. Values below the <77 nL/min cutoff were considered positive. Results with less than 10% internal variability on each nostril were considered reproducible values. Flow calibration for Nitric Oxide Analyzer was completed daily and span calibration monthly using certified Nitric Oxide (NO) in nitrogen gas (N_2_) with a concentration of 2.5 parts per million (ppm).

### 2.4. Statistical Analysis and Power Analysis

The median and interquartile range (25–75%) were calculated for nNO levels for each cohort. Kruskal–Wallis tests were utilized to compare the statistical significance of nNO levels across all cohorts. Wilcoxon signed-rank tests were used to compare nNO levels between paired cohorts (cohort 1 and cohort 4) with continuous and non-parametric variables. Mann–Whitney rank-sum tests were applied to unpaired cohorts with non-parametric continuous variables. Descriptive statistics are presented in means and percentages. An nNO measurement for 10 subjects with PCD (*n* = 10) in cohort 1 provided a confidence level of 99.9%, power of 97%, and a margin of error of ±1%. All statistical analyses were performed using GraphPad Prism version 8.0.0 for IOS, GraphPad Software, San Diego, CA, USA, www.graphpad.com (accessed on 22 July 2022).

## 3. Results

### 3.1. Subjects Characteristics and Demographics

Forty subjects were enrolled in our study and completed the nNO levels measurements per protocol. Each cohort’s demographics and PCD clinical manifestations are presented in (Table 1).

### 3.2. Nasal Nitric Oxide (nNO) Levels Measurements

**Figure 2:**
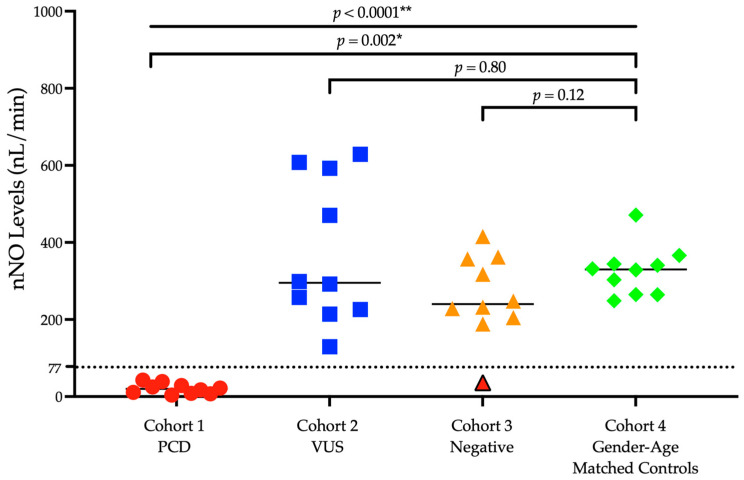
Nasal Nitric Oxide (nNO) Levels in PCD subjects with the *RSPH4A* founder (c.921+3_921+6del (intronic)) pathogenic variant compared with gender–age-matched controls. Cutoff value: 77 nL/min. Subjects in cohorts 2 and 3 compared to cohort 4 were not statistically significantly different. ** Kruskal–Wallis Test, * Wilcoxon Signed-Rank Test. A subject with an nNO value below the cutoff point (red triangle) was identified in cohort 3 with an nNO level of 36.2 nL/min.

Table 2 shows values of nNO for each study cohort as follows cohort 1: PCD confirmed subjects: 19.9 nL/min, cohort 2: PCD Subjects with the presence of genetic variants of unknown significance (VUS): 295.3 nL/min, cohort 3: suspected PCD subjects with negative genetic testing: 240.1 nL/min, and cohort 4: gender-age matched controls: 330.1 nL/min. The levels of nNO were significantly lower across cohorts (*p* = <0.0001 **) and in subjects in cohort 1 as compared with cohort 4, (*p* < 0.002 *) (Figure 2).

## 4. Discussion

This study measured nNO levels in native Puerto Rican patients with the *RSPH4A* (c.921+3_921+6del (intronic)) mutation. The clinical characteristics among cohorts in this study depict the frequency of chronic cough and sinusitis as very common among suspected subjects with PCD in Puerto Rico. As explained in other published studies, bronchiectasis is a critical clinical sign that should raise awareness about PCD as a potential diagnosis [16]. Considering a prevalence of 80% bronchiectasis in the cases with PCD (cohort 1) at the time of diagnosis should be a red flag for PCD. Another clinical feature is hearing loss, which represented 70% of the PCD cases compared with 0% in cohort 2 or 10% in cohort 3. For instance, in a patient with chronic cough, sinusitis, bronchiectasis, and hearing loss in Puerto Rico, medical providers should include PCD as part of the differential diagnosis and early referral to a PCD center.

The measured nNO levels were compared among the following cohorts: subjects with suspected PCD who have one or more VUS, subjects with suspected PCD who had a negative genetic panel, and healthy gender and age-matched controls for cohort 1. The results demonstrate a similar trend as previous publications showing significantly lower nNO levels for PCD patients [11]. The same also applies to our Puerto Rican PCD patients cohort with the founder mutation. We found that the median nNO level for our confirmed PCD cohort was 19.9 nL/min, whereas the gender and age-matched control cohort had a median of 330.1 nL/min. This significant difference points to the potential for nNO to be used as a valuable screening and ancillary diagnostic tool for PCD diagnosis in Puerto Rico and the Caribbean, especially as more awareness is raised within the medical community and more patients with suspected PCD are referred to our center for diagnosis.

For cohorts consisting of patients who have VUS or negative genetic panels, the median new levels were also well above the established diagnostic cutoff of 77 nL/min with 295.3 nL/min and 240.1 nL/min, respectively. This observation further demonstrates that nNO levels were useful to differentiate subjects with only VUS from PCD homozygous patients for the founder mutation. Furthermore, concerning cohort 3, it is likely that not all genes associated with PCD have been described in the literature or included in the standard 42 PCD genes panel used in our study to test for PCD. Our study included a subject in cohort 3 who met the clinical criteria for PCD yet had a negative genetic panel when initially tested for PCD. However, his nNO levels were below the diagnostic cutoff with 36.2 nL/min average across his two visits. Subsequent PCD testing showed microtubular disorganization present on nasal ciliary biopsy on this subject. In cases such as these, a whole-exome gene sequence analysis is a valuable tool to determine if a new mutation or gene is related to PCD. Although an outlier in that specific cohort of our study, this case further illustrates the value of utilizing nNO levels in conjunction with other established ancillary tools to ensure a thorough PCD diagnostic evaluation. While the sample population in our study could be taken as a limitation, a sample size of 10 subjects in cohort 1 with confirmed PCD provided us with enough statistical power to see differences between cohorts. As a limitation, this study analyzed only 42 genes implicated in the PCD from the more than 50 genes currently known to cause the disease.

Our results confirmed that native Puerto Rican PCD patients with the *RSPH4A* (c.921+3_921+6del (intronic)) mutation have nNO levels that are significantly lower as compared with healthy gender-age matched controls. The measurement of nNO levels proved to be a valuable ancillary tool in Puerto Ricans with the founder mutation for PCD diagnosis, especially when paired with the clinical history, physical examination, and other tools such as genetic testing and nasal ciliary biopsy. Although, in general, genetic testing is more widely available, its access in Puerto Rico is still somewhat limited. For instance, the implementation of ancillary non-invasive tools and tests such as nNO assists physicians in the clinical decisions regarding PCD management and treatment. In our study, the nNO levels below 77 nL/min point toward a highly suspected diagnosis which could subsequently be confirmed through further evaluation. Adding this ancillary tool to the diagnostic algorithm in Puerto Rico would be valuable for patients to receive a prompt diagnosis and appropriately characterize our PCD patients with the founder mutation on the island. The implementation of nNO level measurements using state-of-the-art technology supported our accreditation as a PCD center. As the first PCD center in Puerto Rico and the Caribbean, we aim to promote the representation of Hispanics in the PCD registry and collaborate in future clinical trials with the support of the PCD foundation.

## Figures and Tables

**Table 1:** Subject demographics by cohorts and nNO Levels.

	Cohorts				
Characteristics	1	2	3	4	Overall
	PCD	VUS	Negative	Controls	HS
Number of Subjects, (*n*)	10	10	10	10	40
Gender (F), *n* (%)	70	70	40	70	62.5
Age, mean (years)	29	17	16	19	23
Age, ±SD, (years)	19.6	16.3	15.8	18.2	18.1
Bronchiectasis, (%)	80	20	30	0	32.5
Laterality Defects (%)	0	0	10	0	2.5
Chronic Cough *, (%)	100	80	100	0	70
Neonatal Respiratory Distress, (%)	30	20	10	0	15
Chronic Sinusitis, (%)	100	80	60	0	60
Chronic Otitis Media (%)	30	0	10	0	10
Hearing loss, (%)	70	0	10	0	20

F: Female; SD: Standard Deviation. HS: Healthy Subjects; * Persistent, year-round wet cough that starts before 6 months of age.

**Table 2:** Nasal Nitric Oxide (nNO) Levels across all cohorts.

Nitric Oxide Level (nNO)			
	Median nL/min	Interquartile Range 25–75%	*p*-Value
Cohort 1 (PCD)	19.9	[8–31.3]	<0.002 *
Cohort 2 (VUS)	295.3	[222.9–596.2]	0.80
Cohort 3 (Negative)	240.1	[201–358.5]	0.12
Cohort 4 (Gender-age matched controls)	330.1	[264.7–349.4]	-
Overall			<0.0001 **

* Statistical analysis of data between cohorts 1, 2, and 3 as compared with cohort 4 was completed using Wilcoxon signed-rank test. ** Overall statistical significance among cohorts was tested using a Kruskal–Wallis test.

## Data Availability

All data are available upon request through the corresponding author.
